# DCBLD1 Overexpression Is Associated With a Poor Prognosis in Head and Neck Squamous Cell Carcinoma

**DOI:** 10.3389/fimmu.2022.939344

**Published:** 2022-07-01

**Authors:** Ling-ling Fu, Ming Yan, Min-Xian Ma, Yi Luo, Min Shao, Martin Gosau, Reinhard E. Friedrich, Tobias Vollkommer, Hong-chao Feng, Ralf Smeets

**Affiliations:** ^1^ Department of Oral and Maxillofacial Surgery, Guiyang Hospital of Stomatology, Guiyang, China; ^2^ Department of Oral and Maxillofacial Surgery, University Medical Center Hamburg-Eppendorf, Hamburg, Germany; ^3^ Department of Oral and Maxillofacial Surgery, Division of Regenerative Orofacial Medicine, University Medical Center Hamburg-Eppendorf, Hamburg, Germany

**Keywords:** DCBLD1, biomarker, immune infiltrates, prognosis, head and neck squamous cell carcinoma

## Abstract

**Background:**

DCBLD1 is highly expressed in several kinds of cancer and plays a potential prognostic factor. However, the prognostic value and immune infiltration in head and neck squamous cell carcinoma remain unclear and need further research.

**Materials and Methods:**

DCBLD1 expression and clinical information were obtained from the Cancer Genome Atlas (TCGA) database. The mRNA level in cell lines (SCC25 and CAL27) and gingival fibroblasts were detected using quantitative PCR. Cox regression analysis was used to evaluate the prognostic values of DCBLD1 and clinical data in HNSCC. A nomogram was also established to predict the impact of DCBLD1 on prognosis based on Cox multivariate results. The methylation level of DCBLD1 in HNSC and its prognosis were analyzed in UALACN and MethSurv. Finally, the potential biological functions of DCBLD1 were investigated using gene set enrichment analysis (GSEA) and single-sample GSEA (ssGSEA).

**Results:**

The mRNA and protein expression levels of DCBLD1 were highly expressed in HNSCC tissue and cell lines. The Cox analyses demonstrate that highly expressed DCBLD1 is an independent prognosis marker (*p* < 0.05). ROC curve analysis showed the performance of DCBLD1 (area under the ROC curve: 0.948, sensitivity: 93.2%, specificity: 84.7%). The methylation was increased in HNSCC patients compared with normal subjects (*p* < 0.05) and was associated with poor prognosis at sites cg27642470 and cg21104965. Additionally, DCBLD1 expression is poorly associated with immune cell infiltration and immunological checkpoints PD-L1 and TIM-3.

**Conclusion:**

In head and neck squamous cell carcinoma, DCBLD1 is overexpressed, associated with poor patient prognosis. The detailed underlying mechanism merits further research.

## Introduction

Head and neck cancer is a broad term that encompasses epithelial malignancies in the oral cavity, hypopharynx, nasopharynx, and larynx, mainly consisting of squamous cell carcinomas [head and neck squamous cell carcinoma (HNSCC)]. HNSCC is the sixth most frequent cancer worldwide, with an incidence of over 830,000 new cases annually (1). Although surgical and comprehensive treatment techniques over the last few decades have improved the quality of life and longevity, the prognosis of HNSCC patients remains poor due to local recurrence and distant metastasis.

The clinical significance of the mRNA expression has been previously described in several human cancer, including HNSCC (2). In addition, they play an important role in various physiological and pathological processes. For example, overexpression of SEC61G and HMMR has been associated with poor prognosis in HNSCC (3, 4).

DCBLD1, a transmembrane protein with extracellular CUB (an extracellular domain of approximately 110 residues that is found in functionally diverse, mostly developmentally regulated proteins), LCCL (including 94–97 amino acids, non-cytoplasmic but the function is undefined), and F5/8 type C domains 8, is a type I transmembrane protein (A–D) that binds to semaphorins (5, 6) belonging to the DCBLD family, and is rarely studied and poorly characterized (5). DCBLD2 is another member of the DCBLD family and has been identified as a prognostic marker in HNSCC (7). It was reported that DCBLD2 was associated with lung cancer metastasis and involved cell growth and proliferation. DCBLD1 and DCBLD2 have similar amino acids, meaning that they may have related functions (5, 8). The domain structure of DCBLD also closely resembles neuropilins harboring two CUB and discoidin domains (9). Additionally, both act as co-receptors for class 3 semaphorins and growth factors in axon guidance and angiogenesis (10). Neuropilin proteins are a class of transmembrane receptors that express on the surface of a large number of tumor cells and are widely involved in regulating tumor angiogenesis. Previous research showed that neuropilins could regulate multiple biological behaviors of tumor cells by acting as an oncogene, such as proliferation, migration, invasion, and apoptosis (9–11). In non-smoking women, a susceptible locus for lung cancer in the DCBLD1 gene was noted (12), suggesting the tumorigenic potential of DCBLD1 for the first time. Wang et al. (13) found that the knockout of DCBLD1 gene significantly affected the tumor formation ability of non-small cell lung cancer cells (A549) and also suppressed cellular proliferation (A549 and NCI-H1299). Cardin et al. (14) reported a potential association of DCBLD1 with the prognosis of HNSCC by studying the expression of rs6942067, a single-nucleotide polymorphism (SNP) located upstream of the DCBLD1 gene. However, there are no relevant studies about the role of DCBLD1 in the tumor microenvironment and prognosis in HNSCC.

In the present study, the RNA-sequencing data of DCBLD1 were downloaded from The Cancer Genome Atlas (TCGA) database. We determined the differential expression of DCBLD1 in pan-cancer and HNSCC from 546 patients. Culturing two HNSCC lines, SCC25 and CAL27, and human gingival fibroblasts (HGFs), the mRNA levels for DCBLD1 were quantified using real-time PCR among different groups. Subsequently, we detected the relationship between DCBLD1 expression with clinical characteristics and prognostication and established a predictive nomogram to investigate the functional role of DCBLD1 in HNSCC using functional enrichment analysis, protein interaction network, immune infiltration, mutation, and methylation.

## Materials and Methods

### Data Acquisition

Level 3 HTSeq-FPKM (fragments per kilobase of transcript per million mapped reads) data of HNSCC patients, including 44 normal and 502 tumor cases, were downloaded from the TCGA data portal (http://tcga-data.nci.nih.gov/tcga/), then were transformed into transcripts per million reads (TPM) and log2-transformed for subsequent analyses. Gene expression data were divided into high and low groups according to the median expression levels of DCBLD1. As TCGA was an open public database, obtaining relevant information did not require additional ethics approval.

### Analysis of Differentially Expressed Genes Between High and Low DCBLD1 Expression Groups in HNSCC Patients

Expression profiles (HTSeq-Counts) were compared between the high DCBLD1 expression group and the low DCBLD1 expression group to identify differentially expressed genes (DEGs) using Wilcoxon rank-sum test (15) in the R language-related software, DESeq2 (version 1.26.0). Differences with a |log2 fold change| > 2 and adjusted *p*-value < 0.05 were considered threshold values for identifying DEGs.

### Enrichment Analysis

Gene Ontology (GO) enrichment and Kyoto Encyclopedia of Genes and Genomes (KEGG) pathway analyses of the top 200 DCBLD1-related genes were performed by the “ClusterProfiler” package (16) and visualized by the “ggplot2” package. In addition, the protein–protein interaction network of DCBLD1 co-expressed genes was visualized by STRING (http://string-db.org; version 11.5) with a minimum level of confidence >0.4 to analyze the functional interactions among proteins (17).

### Cell Culture

Two HNSCC cell lines (CAL27 and SCC25) were purchased from the American Type Culture Collection (ATCC). Moreover, gingival fibroblast cells were cultured from normal tissues.

### RNA Isolation and qPCR

SCC25, CAL27, and gingival fibroblast cells were harvested using a cell scraper after washing 3 times with phosphate-buffered saline (PBS). RNA was extracted through the following steps: lysing cells in Trizol reagent (Cat No. 15596-026, Life Technologies, Carlsbad, CA, USA), followed by extracting RNA in trichloromethane, and then precipitating it in isopropanol, and finally resuspending it in RNase-free water. The RNA concentration and purity levels were determined using a Nanodrop2000 Spectrophotometer (Thermo Fisher Scientific, Waltham, MA, USA). Total RNA (2.5 µg) was subjected to cDNA synthesis using a qScript cDNA SuperMix (Quanta Biosciences, Beverly, MA, USA) through the following consequent cycles: firstly at 25°C for 5 min, followed by 42°C for 30 min and finally at 85°C for 5 min. A real-time PCR was performed to determine the mRNA levels of DCBLD1 and GAPDH using SYBR Green Master MIX (ABI, Vernon, CA, USA). Real-time PCR results were calculated using the 2^−ΔΔCq^ method.

### Gene Set Enrichment Analysis

Gene set enrichment analysis (GSEA) (18) is used to access the concordance between our data and *a priori*-defined gene set. This study performed GSEA with the R package ClusterProfiler (3.14.3) (16) to elucidate the significant function and pathway differences between the high and low DCBLD1 expression groups. Each analysis procedure was repeated 5,000 times. A function or pathway term with adjusted *p*-value < 0.05, false discovery rate (FDR) < 0.25, and normalized enrichment score |NES| > 1 was considered to be statistically significant enrichment.

### Analysis of DCBLD1 Mutation, Methylation, and Prognosis

The mutation data of DCBLD1 and its association with overall survival (OS) were analyzed from the cBioPortal (https://www.cbioportal.org/) web platform (19). Moreover, DCBLD1 methylation data and its prognostic value were downloaded from UALCAN (http://ualcan.path.uab.edu/) and MethSurv (https://biit.cs.ut.ee/methsurv/) online tools (20).

### Immune Infiltration Analysis by Single-Sample GSEA

Immune infiltration analysis of HNSCC samples was performed by the single-sample GSEA (ssGSEA) (21) method using the GSVA package in R for 24 types of immune cells, namely, neutrophils, mast cells, eosinophils, macrophages, natural killer (NK) cells, CD56dim NK cells, CD56bright NK cells, dendritic cells (DCs), immature DCs (iDCs), activated DCs (aDCs), plasmacytoid DCs (pDCs), T cells, CD8^+^ T cells, T helper (Th) cells, Th1 cells, Th2 cells, Th17 cells, T follicular helper cells, regulatory T cells (Treg), central memory T cells (Tcm), effector memory T cells (Tem), gamma delta T cells (Tgd), cytotoxic cells, and B cells. The relative enrichment score of each immunocyte was calculated using the reported signature genes for the 24 types of immunocytes and the gene expression profile for each tumor sample. Furthermore, the relationship between DCBLD1 and immune cell markers was analyzed by TIMER (http://timer.comp-genomics.org/) online tools (22).

### Statistical Analysis

All data analyses and plots were performed using R language (Version 3.6.3) and visualized by R package ggplot2. Wilcoxon rank-sum test and Wilcoxon signed-rank test were used to analyze the expression of DCBLD1 in unpaired and paired samples, respectively. In addition, ROC analysis and the frequently used method for binary assessment were conducted using the pROC package (1.17.0.1) to assess the diagnostic capability of DCBLD1 in head and neck cancer. The computed AUC value from 0.5 to 1 indicates the discriminative potential from 50% to 100%. The prognostic data were obtained from Cell (23), while the survival package (3.2-10) for Cox regression analyses and the Kaplan–Meier method were used to evaluate prognostic factors. In all tests, *p*-value < 0.05 was considered statistically significant.

## Results

### DCBLD1 mRNA Is Overexpressed in HNSCC

**Figure 1:**
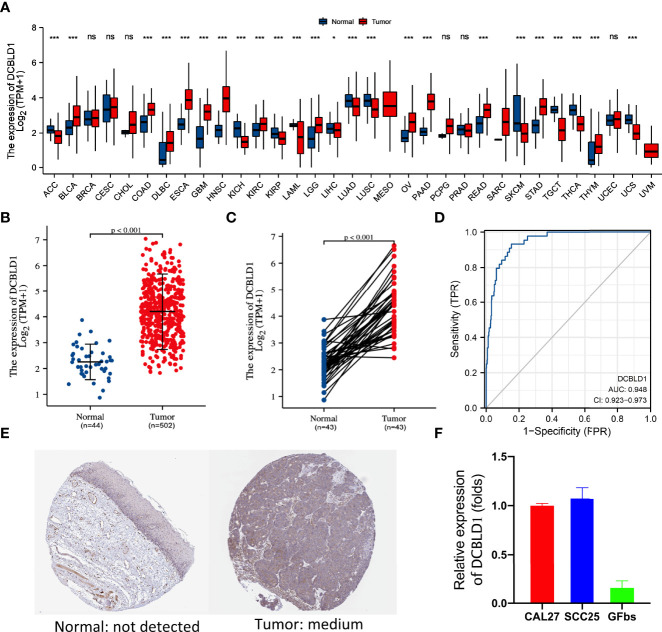
DCBLD1 expression in HNSCC patients. **(A)** DCBLD1 expression level in pan-cancer analysis from the TCGA database. **(B)** DCBLD1 expression in normal (*n* = 44) and HNSCC (*n* = 502) patients. **(C)** DCBLD1 expression in cancer samples and matched normal tissues. **(D)** Receiver operating characteristic (ROC) curve analysis of DCBLD1 in HNSCC. **(E)** Immunohistochemical analysis of DCBLD1 from the Human Protein Atlas. **(F)** The level of DCBLD1 mRNA expression in CCL27, SCC25, and gingival fibroblasts.

The TCGA database was utilized to identify the DCBLD1 mRNA expression in different cancers, which was highly expressed in 12 of the 33 tumor types, as shown in **Figure 1A**. DCBLD1 mRNA expression was significantly higher in 502 head and neck cancer patients than those in 44 non-cancer subjects (*p* < 0.001, **Figure 1B**) and was also higher in 43 tumor tissue paired with pericarcinous tissue (*p* < 0.001, **Figure 1C**). The immunohistochemistry was performed to validate the expression of DCBLD1 in normal and HNSCC tissue at the protein level, which was downloaded from the HPA database (**Figure 1E**). To further analyze the significance of DCBLD1 expression, qPCR results were shown in HNSCC cell lines (SCC25 and CAL27) and gingival fibroblasts. (**Figure 1F**). A relatively high AUC value showed an excellent predictive power of DCBLD1 expression to identify tumors from normal tissue (AUC = 0.948, **Figure 1D**).

### Identification of Differentially Expressed Genes Between the High and Low DCBLD1 Expression Groups

**Figure 2:**
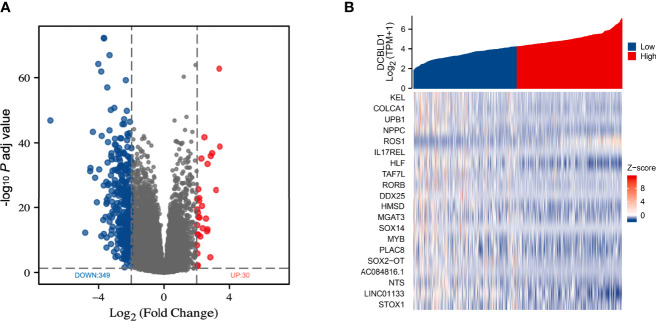
Differentially expressed genes in HNSCC with high and low DCBLD1 expressions. **(A)** Volcano plot of DEGs; the downregulated genes were in blue (*n* = 349), and the upregulated genes were in red (*n* = 30). **(B)** Heatmap of the top 20 DEGs between high and low DCBLD1 groups.

TCGA data were analyzed using the DSEeq2 package in R (|log FC| > 2, adjusted *p*-value < 0.05), and a total of 379 DEGs were found between the groups with high and low DCBLD1 expressions, including 349 downregulated genes in blue color and 30 upregulated genes in red color (**Figure 2A**). In addition, the top 20 DEGs are shown as a heatmap (**Figure 2B**).

### Association of DCBLD1 mRNA With Clinical Characteristics

The association between DCBLD1 mRNA expression and clinical parameters in HNSCC was assessed (**Table 1**). The high DCBLD1 expression was associated with lymph node neck dissection (*p* < 0.05). However, no statistically significant relationship was found between DCBLD1 mRNA expression, age, clinical stage, and tumor grade.

**Table 1:** DCBLD1 expression in HNSCC patients with different clinical parameters.

Characteristics	DCBLD1 mRNA expression		*p*
	Low (*n* = 250)	High (*n* = 250)	
**Age**			0.755
≤60	120 (24%)	124 (24.8%)	
>60	130 (26.1%)	125 (25.1%)	
**T stage**			0.430
T1	17 (3.5%)	16 (3.3%)	
T2	80 (16.5%)	63 (13%)	
T3	62 (12.8%)	68 (14%)	
T4	85 (17.5%)	94 (19.4%)	
**N stage**			0.366
N0	122 (25.5%)	117 (24.5%)	
N1	35 (7.3%)	45 (9.4%)	
N2	81 (16.9%)	71 (14.9%)	
N3	2 (0.4%)	5 (1%)	
**M stage**			0.684
M0	237 (49.9%)	233 (49.1%)	
M1	2 (0.4%)	3 (0.6%)	
**Clinical stage**			0.637
Stage I	8 (1.6%)	11 (2.3%)	
Stage II	45 (9.3%)	50 (10.3%)	
Stage III	56 (11.5%)	46 (9.5%)	
Stage IV	136 (28%)	134 (27.6%)	
**Lymph node neck dissection**			0.025*
No	55 (11.1%)	35 (7%)	
Yes	193 (38.8%)	214 (43.1%)	

### High DCBLD1 Expression Is Associated With Poor Prognosis in HNSCC

To confirm the correlation of DCBLD1 expression with OS and disease-specific survival (DSS) in patients, the influencing prognostic factors were identified by Cox regression analysis. Multivariate analyses revealed that DCBLD1 was an independent prognostic factor for OS in patients with HNSCC (HR = 0.631, 95% CI: 0.410–0.971, *p* = 0.036). Furthermore, primary therapy outcome (HR = 0.209, 95% CI: 0.125–0.350, *p* < 0.001), lymphovascular invasion (HR = 1.657, 95% CI: 1.025–2.680, *p* = 0.039), and radiation therapy (HR = 0.534, 95% CI: 0.327–0.871, *p* = 0.012) were also an independent prognostic factor for OS in patients (**Table 2**). DSS could better reflect the disease-specific clinical benefits. Based on the multivariate analysis of prognostic factors, DCBLD1 was also an independent prognostic factor of DSS (HR = 0.502, 95% CI: 0.298–0.845, *p* = 0.009, **Supplementary Table 1**).

**Table 2:** Association of clinicopathological characteristics with overall survival using univariate or multivariate Cox regression analysis.

Characteristics	Total (*N*)	Univariate analysis		Multivariate analysis	
		Hazard ratio (95% CI)	*p*-value	Hazard ratio (95% CI)	*p*-value
Age (≤60 vs. >60)	501	1.252 (0.956–1.639)	0.102		
Gender (Female vs. Male)	501	0.764 (0.574–1.018)	0.066	0.975 (0.611–1.556)	0.915
Clinical stage (Stage I and Stage II vs. Stage III and Stage IV)	487	1.217 (0.878–1.688)	0.238		
T stage (T1 and T2 vs. T3 and T4)	486	1.245 (0.932–1.661)	0.137		
N stage (N0 vs. N1 and N2 and N3)	479	1.263 (0.964–1.653)	0.090	1.603 (0.994–2.585)	0.053
M stage (M0 vs. M1)	476	4.745 (1.748–12.883)	**0.002**	2.041 (0.241–17.265)	0.513
Primary therapy outcome (PD and SD and PR vs. CR)	417	0.182 (0.124–0.268)	**<0.001**	0.209 (0.125–0.350)	**<0.001**
Histologic grade (G1 vs. G2 and G3)	480	1.592 (1.025–2.474)	**0.039**	1.275 (0.590–2.756)	0.537
Lymphovascular invasion (No vs. Yes)	340	1.699 (1.211–2.384)	**0.002**	1.657 (1.025–2.680)	**0.039**
Lymph node neck dissection (No vs. Yes)	498	0.731 (0.526–1.016)	0.062	0.667 (0.289–1.543)	0.344
DCBLD1(High vs. Low)	501	0.707 (0.540–0.925)	**0.012**	0.631 (0.410–0.971)	**0.036**
Radiation therapy (No vs. Yes)	440	0.613 (0.452–0.831)	**0.002**	0.534 (0.327–0.871)	**0.012**
Race (White vs. Black or African American and Asian)	485	1.470 (0.973–2.220)	0.067	1.256 (0.654–2.409)	0.494

Bold values P<0.05

**Figure 3:**
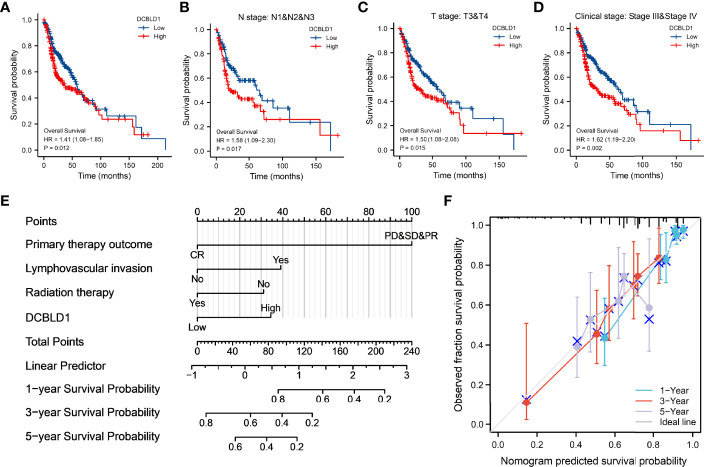
The association of DCBLD1 expression and prognosis in HNSCC. **(A–D)** Overall survival. **(E)** Nomogram integrates DCBLD1 and other prognostic factors in HNSCC from the TCGA data. **(F)** The calibration plot of the nomogram.

Correlations between DCBLD1 expression and prognosis in HNSCC patients were shown in the Kaplan–Meier plot. Patients with higher DCBLD1 expression had a shorter OS time in different subgroups (*p* < 0.05, **Figures 3A–D**).

The nomogram plot showed the prognosis of DCBLD1 and the relative clinical situation. Based on the multivariate Cox analysis, a nomogram was assigned to the clinical characteristic of a point, and the sum of the points was awarded as the total points. The probability of DCBLD1 survival at 1, 3, and 5 years can be determined using the absolute point axis down to the outcome axis (**Figure 3E**). The calibration curve evaluated the nomogram’s performance of DCBLD1. The C-index of nomogram was 0.720 with 1,000 bootstrap replicates (95% CI: 0.693–0.747). The bias-corrected line in the calibration plot was close to the 45° line, which is the ideal curve (**Figure 3F**).

### Function and Pathway Enrichment Analysis

**Figure 4:**
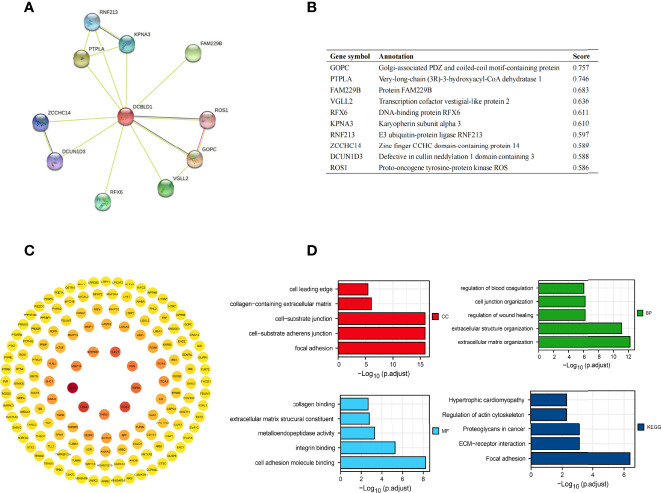
Protein–protein interaction network and function enrichment in HNSCC. **(A, B)** DCBLD1 interaction protein plot and description. **(C)** PPI network of DCBLD1 and its co-expression genes. **(D)** GO and KEGG enrichment analyses of DCBLD1-related genes.

The top ten related proteins to DCBLD1 were established by STRING (**Figures 4A, B**). A total of 197 correlated genes with DCBLD1 in the TCGA-HNSCC database were evaluated (|cor Pearson| > 0.5, *p*-value < 0.05), including 193 positively correlated genes and 4 negatively correlated genes. The PPI network of those 197 co-expressed genes was established in STRING and Cytoscape (**Figure 4C**). GO and KEGG enrichment analyses investigated the functional significance of DCBLD1 co-expressed genes in HNSCC. The top 20 enriched items, including cellular components, molecular function, biological process, and KEGG, are shown in **Figure 4D**.

### Gene Set Enrichment Analysis

**Figure 5:**
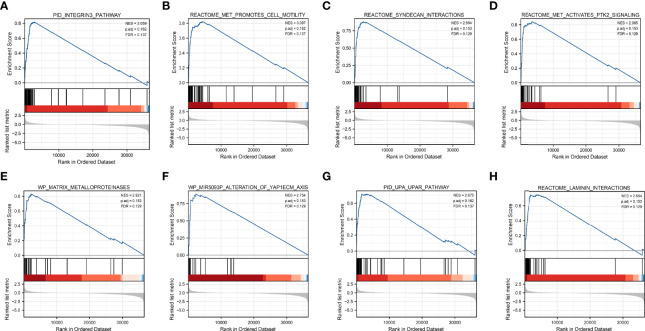
Gene set enrichment analysis of DCBLD1 in HNSCC. **(A)** Integrins 3 pathway. **(B)** Met promotes cell motility. **(C)** Syndecan interactions. **(D)** Met activates PTK2 signaling. **(E)** Matrix metalloproteinases. **(F)** MIR5093P alteration of the YAP1ECM axis. **(G)** UPA UPAR pathway. **(H)** Laminin interactions. NES, normalized enrichment score; FDR, false discovery rate.

Based on significant differences (adjusted *p*-value < 0.05, FDR < 0.25), GSEA was used to identify signaling pathways associated with HNSCC between the high and low DCBLD1 expression groups, including 102 positive regulation pathways and 74 negative regulation pathways. The most significantly enriched pathways were the integrins 3 pathway, met promotes cell motility, syndecan interactions, met activates PTK2 signaling, matrix metalloproteinases, MIR5093P alteration of YAP1ECM axis, UPA UPAR pathway, and laminin interactions (**Figure 5**).

### Bioinformatical Analysis

**Figure 6:**
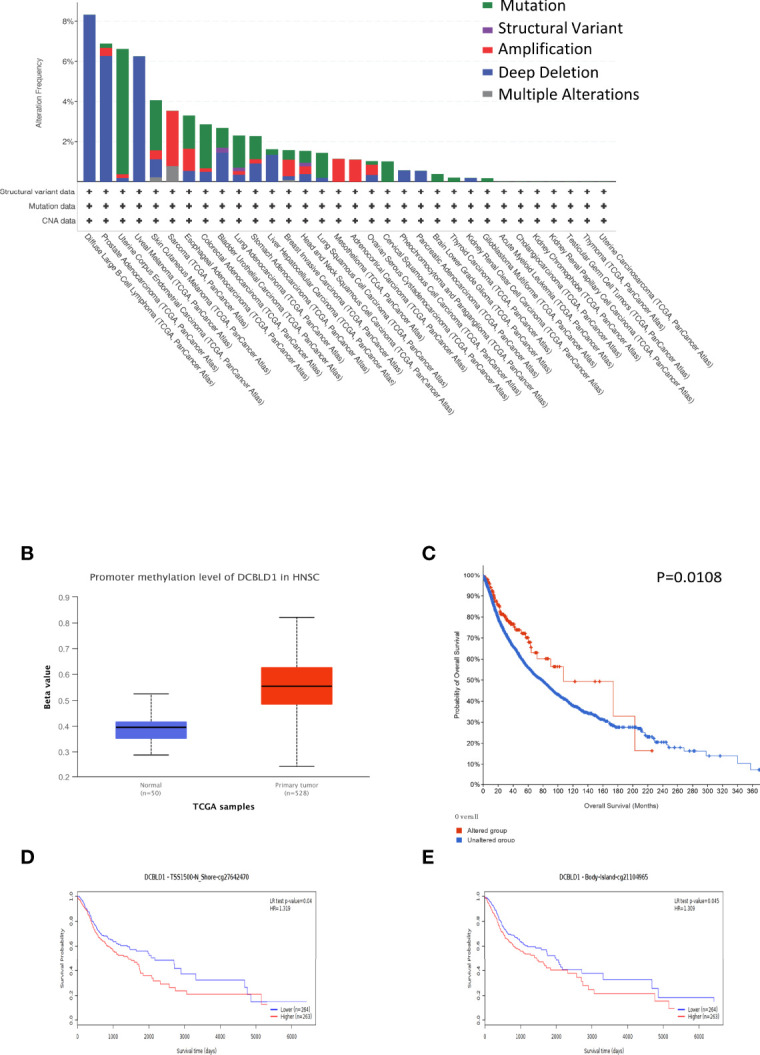
The gene mutation and methylation of DCBLD1 in HNSCC. **(A)** DCBLD1 mutation in pan-cancer. **(B)** The methylation level of DCBLD1 in HNSCC. **(C)** The association of DCBLD1 alteration and overall survival of HNSCC. **(D, E)** The Kaplan–Meier survival of the promoter methylation of DCBLD1.

The correlation between DCBLD1 expression and its mutation in pan-cancer was analyzed using cBioPortal, as revealed in **Figure 6A**. In 523 HNSCC cases, the genetic alteration was found in eight patients, and the mutation rate was 1.53%. Moreover, the results revealed that genetic alteration in HNSCC was associated with superior OS of HNSCC, implying that the genetic mutation of DCBLD1 could also affect HNSCC patients’ prognosis. The methylation level of DCBLD1 in HNSCC was examined by UALCAN based on TCGA. The methylation was higher in the high DCBLD1 expression group (**Figure 6B**). MethSurv analysis showed that patients with high DCBLD1 methylation had a worse OS than patients with low DCBLD1 methylation (**Figure 6C**). The 2 CpG sites are shown in **Figures 6D, E**.

### The Correlation Between DCBLD1 and Immune Infiltration in HNSCC

**Figure 7:**
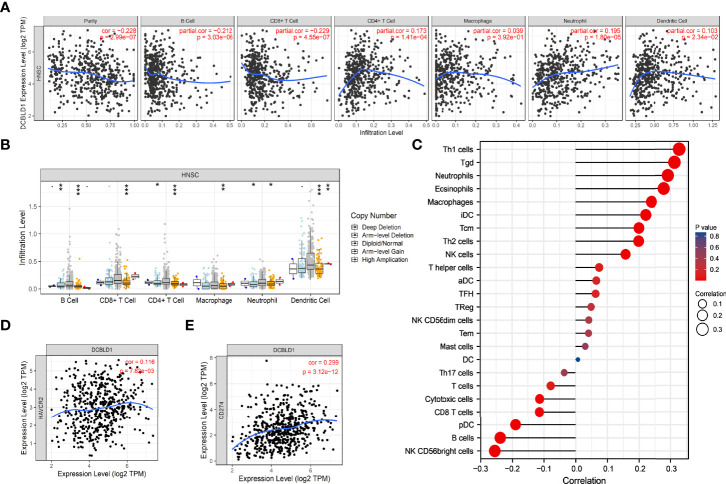
The immune infiltration of DCBLD1 expression in HNSCC. **(A)** Association between DCBLD1 expression and the infiltration of immune cells. **(B)** The visualization between DCBLD1 expression and SCNA in HNSCC from the TCGA data. **(C)** Correlations between 24 immune cells and DCBLD1 expression. **(D)** The correlation of DCBLD1 and HAVCR2 (TIM-3). **(E)** The correlation of DCBLD1 and CD274(PD-L1).

The association between DCBLD1 expression and immune cell infiltration was visualized using ssGSEA and the TIMER software. DCBLD1 expression was poorly correlated with the abundance of eosinophils, iDCs, macrophages, neutrophils, NK cells, Tcm, Tgd, Th1 cells, and Th2 cells, and was negatively correlated with the abundance of B cells, CD8 T cells, cytotoxic cells, NK CD56bright cells, pDCs, and Th17 cells (**Figures 7A, C**). The correlation of DCBLD1 and immune cell markers analyzed by TIMER is shown in **Table 3**, which demonstrates that high DCBLD1 expression was associated with most immune cell markers but also with a poor correlation. Previous studies showed that higher B-cell and CD8^+^ T infiltration were associated with poor prognosis. The SCNA module compares tumor infiltration relationships among tumors with different somatic copy number variations for DCBLD1 (**Figure 7B**).

**Table 3:** Correlation of DCBLD1 and immune cell markers analyzed by TIMER.

Description	Gene markers	None		Purity	
		cor	*p*	cor	*p*
CD8^+^ T cell	CD8A CD8B	−0.061 −0.131	1.61e−01 2.76e−03^**^	−0.111 −0.178	1.37e−02^*^ 7.46e−05^***^
T cell	CD3D CD3E CD2	−0.122 −0.065 −0.063	5.31e−03^**^ 1.38e−01 1.51e−01	−0.181 −0.125 −0.115	5.40e−05^***^ 5.52e−03^**^ 1.06e−02^*^
B cell	CD19 CD79A	−0.271 −0.276	3.17e−10^***^ 1.34e−10^***^	−0.331 −0.328	4.64e−14^***^ 8.03e−14^***^
Monocyte	CD86 CD115(CSF1R)	0.206 0.178	2.20e−06^***^ 4.54e−05^***^	0.166 0.128	2.14e−04^***^ 4.39e−03^**^
M1 Macrophage	INOS(NOS2) IRF5 COX2(PTGS2)	−0.283 −0.109 0.038	4.54e−11^***^ 1.28e−02^*^ 3.82e−01	−0.26 −0.112 0.094	4.93e−09^***^ 1.26e−02^*^ 3.74e−02^*^
M2 Macrophage	CD163 VSIG4 MS4A4A	0.199 0.194 0.173	4.53e−06^***^ 8.2e−06^***^ 7.02e−05^***^	0.134 0.132 0.113	2.82e−03^**^ 3.32e−03^**^ 1.23e−02^*^
Th1	T-bet (TBX21) STAT4 STAT1 IFN-γ(IFNG) TNF-α(TNF) IL12A IL12B	−0.069 0.152 0.298 −0.022 0.173 −0.113 −0.051	1.16e−01 5.09e−04^***^ 3.26e−12^***^ 6.19e−01 7.21e−05^***^ 9.67e−03^**^ 2.44e−01	−0.113 0.117 0.259 −0.06 0.174 −0.092 −0.091	1.21e−02^*^ 9.16e−03^**^ 5.79e−09^***^ 1.86e−01 1.00e−04^***^ 4.24e−02^*^ 4.28e−02^*^
Th2	GATA3 STAT6 STAT5A IL13	0.156 0.015 −0.094 0.032	3.4e−04^***^ 7.4e−01 3.17e−02^*^ 4.61e−01	0.126 0.033 −0.104 0.005	5.16e−03^**^ 4.67e−01 2.08e−02^*^ 9.09e−01
Th17	STAT3 IL17A	−0.004 −0.081	9.33e−01 6.35e−02^*^	0.005 −0.087	9.09e−01 5.31e−02^*^
Treg	FOXP3 CCR8 STAT5B TGFβ(TGFB1)	0.091 0.162 0.139 0.512	3.73e−02^*^ 1.99e−04^***^ 1.45e−03^**^ 2.98e−36^***^	0.06 0.142 0.146 0.487	1.81e−01 1.58e−03^**^ 1.17e−03^**^ 1.02e−30^***^

*p < 0.05; **p < 0.01; ***p < 0.001.

Solid tumors, especially melanoma and renal cancer, have significantly benefited from immunotherapy, especially from immune checkpoint inhibitors (ICIs), which also offer potential predictive and therapeutic strategies for R/M HNSCC. In 2014, FDA approved using nivolumab and pembrolizumab, targeting PD-1/PD-L1 interaction. Both demonstrated better survival and response rates in clinical trials CheckMate-141 and KEYNOTE-012. TIM-3 and PD-L1 play an important role in tumor immune escape, which were also recognized as immune checkpoints in HNSCC. The expression of DCBLD1 was positively related to those immune checkpoints based on the HNSCC-TCGA database (**Figures 7D, E**).

## Discussion

This study analyzed DCBLD1 expression in pan-cancer using the TCGA data and found upregulated expression in BLCA, COAD, DLBA, ESCA, GBM, HNSC, KIRC, LAML, LGG, LUSC, OV, PAAD, READ, STAD, and THYM as compared to healthy individuals. Subsequently, HNSCC was focused, and a higher expression of DCBLD1 was demonstrated in cancer tissues than in normal tissues (*p* < 0.001). Combining HPA data and qPCR results, it was found that DCBLD1 proteins were moderately expressed in OSCC tissues but were absent in normal oral tissues. SCC25 and CAL27 cancer cell lines are derived from human OSCC commonly used in HNSCC studies (24), while gingival fibroblasts originated from the gingival tissues resected in normal people after the removal of impacted teeth. The qPCR results indicated that the expression of DCBLD1 mRNA in OSCC cells was higher than that in normal gingival fibroblasts, which further suggested that high DCBLD1 expression might associate with the development or progression of HNSCC. Previous studies about DCBLD1 were scarce, so the carcinogenic role of DCBLD1 remains unclear. Concurrently, Wang et al. (13) found that DCBLD1 was associated with tumor formation by knocking down this gene. Furthermore, the AUC score reached a value up to 0.948, demonstrating good diagnostic power of DCBLD1 expression toward HNSCC (25). Previous studies also showed the associations of the DCBLD1 expression with lung cancer and breast cancer. All these findings suggest the oncogenic potential of DCBLD1.

The present study also revealed the significant prognostic value of DCBLD1 in the survival outcome of HNSCC. According to Kaplan–Meier curves, high DCBLD1 expression predicted a shorter OS time in patients with HNSCC. Consistent findings were also demonstrated in subgroup analysis. This also implied the potential of DCBLD1 as an oncogene. Additionally, DCBLD1 was identified as an independent prognostic factor for the OS and DSS of HNSCC using univariate and multivariate Cox regression analyses. Collectively, DCBLD1 might be a potential therapeutic target for treating HNSCC. The nomogram is a reliable, effective tool for cancer prognosis (26). Here, a nomogram combining DCBLD1 expression, primary therapy outcome, lymphovascular invasion, and radiation therapy was established and identified to be effective in the prognosis of HNSCC according to the calibration curve. We noted that high DCBLD1 expression accounted for most of the contribution to the nomogram in the prognosis of HNSCC, suggesting that DCBLD1 is a potent prognostic factor. In addition, the present study also explored the role of mutation and promoter methylation of the DCBLD1 gene in HNSCC prognosis. Mutations in the DCBLD1 gene were associated with good survival outcomes. It was also noted that DCBLD1 gene promoter methylation was much more prevalent in HNSCC tissue than in normal tissue. The current study indicated that methylation at cg27642470 and cg21104965 predicted a poor prognosis.

The tumor microenvironment is significantly associated with tumor growth, development, and prognosis (27). Inflammatory cells, essential components of the tumor microenvironment (27, 28), can advance the degradation of the extracellular matrix (ECM), potentiate angiogenesis and tissue remodeling, and increase cellular migration. Koneva et al. (29) analyzed lymphocyte subtypes in HNSCC and reported many tumor-infiltrating lymphocytes. Another retrospective study about HNSCC showed that a high infiltration abundance of CD4^+^ and CD8^+^ T cells indicated a higher risk of death (30), demonstrating that upregulated infiltration of the two immune cell types was prognostic for the recurrence-free survival in HNSCC patients. Here, we found that the expression of DCBLD1 was positively associated with the infiltration abundance of eosinophils, iDC, macrophages, neutrophils, NK cells, Tcm, Tgd, Th1 cells, and Th2 cells in the tumor microenvironment and negatively associated with the infiltration abundance of B cells, CD8 T cells, cytotoxic cells, NK CD56 bright cells, pDCs, and Th17 cells. However, the correlation was poor in all correlations. This indicated that DCBLD1 is not associated with immune cell infiltration in head and neck cancer.

The copy number of the DCBLD1 gene was also observed to play a role in immune infiltration in HNSC. According to the data retrieved from the GEPIA database, DCBLD1 gene expression was positively correlated not only with the expression of immune checkpoints PD-L1 and TIM-3 but also with a poor association.

Immunotherapy is a step forward from traditional chemoradiotherapy based on interactions between tumor cells and the immune microenvironment (31, 32).

In the tumor microenvironment, infiltrating immune cells experience functional impairment by expressing multiple inhibitory signals on the cell surface, such as PD-1/PD-L1, CTLA-4, TIGIT, and TIM-3, leading to tumor immunosuppression. These inhibitory signals are also known as immune checkpoints (33). PD-1/PD-L1 is among the earliest used in treating advanced melanoma (34). The tumor cell-intrinsic PD-1 receptor is an important modulator of immune responses and primarily suppresses them, negatively regulating T cells and immune response. PD-1:PD-L1 binding inhibits T-cell activation, decreasing proliferation and effector cytokine production. The FDA has approved pembrolizumab and nivolumab for clinical treatment. In the clinical tests, pembrolizumab combined with chemoradiotherapy compared to chemoradiotherapy alone improved tumor response and progression-free survival in patients with recurrent/metastatic (R/M) HNSCC. Close to 20% of them survive more than 4 years. With nivolumab, patients had a longer OS than those with standard treatment (7.5 months versus 5.1 months). PD-L1 overexpression was demonstrated in 18%–96% of OSCC patients and associated with lymph node metastasis (35). Zhang et al. believed that PD-L1 overexpression predicted a higher risk of lymph node metastasis (36). Other reports also revealed that PD-L1 overexpression was correlated with the cytokine-induced immune silence of lymphocytes or regional recurrence. These studies suggest that PD-L1 is intimately associated with metastasis to peri-tumor lymph nodes (37). TIM-3 expresses on T cells, NK cells, and some antigen-presenting cells. It is believed to be positively associated with lymph node metastasis and tumor recurrence. Previous research found that high expression of TIM-3 led to effector T-cell depletion, which might cause an ineffective anti-tumor immune response and tumor clearance, resulting in metastasis and recurrence (38). The current study identified a poor association between DCBLD1 expression and PD-L1 and TIM-3.

Following the GSEA study, the integrin pathway was the most significant (39). It has been established that the integrin pathway will be triggered upon binding with ECM, accompanied by activation of downstream actin reorganization and MAPK signaling cascade (40). In HNSCC, the integrin pathway is involved in disease progression, tumor stem cells, and radio-resistance (41, 42). We also noted that GO annotations related to DCBLD1 gene included focal adhesion, cell-substrate junction, ECM organization, cell adhesion molecule binding, and other biological processes involved in ECM and cell adhesion. This suggests the important role of DCBLD1 in tumor progression and migration.

However, the role of DCBLD1 in developing SCC of the oral cavity and other sites of the head and neck region has not yet been fully elucidated. The potential of DCBLD1 as an oncogene in HNSCC has not yet been demonstrated and is a matter of future studies. More *in vivo* and *in vitro* experiments are needed to explore this issue further. We plan to conduct cytological experiments in our future experiments.

## Conclusion

For the first time, this study reported the effects of the DCBLD1 gene on the tumor microenvironment and prognosis in HNSC. Our study suggests that DCBLD1 is a potential prognostic factor for survival outcomes but is not associated with immune cell infiltration. DCBLD1 may also play a role in HNSC progression and metastasis *via* the integrin pathway and cell adhesion. DCBLD1 is expected to be a novel diagnostic and prognostic factor for HNSCC.

## Data Availability Statement

Publicly available datasets were analyzed in this study. This data can be found here: TCGA.

## Author Contributions

L-lF: conceived the study, supervised the experiments, and drafted the manuscript. MY: conceived the study, supervised the experiments, and drafted the manuscript. M-XM: data evaluation and manuscript preparation. YL and MS: data evaluation and manuscript preparation. MG, RF, TV, and RS: analyzed the data and revised the manuscript. H-cF: conceived the study, designed the data evaluation, and prepared the manuscript. All authors contributed to the article and approved the submitted version.

## Funding

This study was supported in part by grants from the Guiyang Municipal Health Bureau Fund for Science and Technology projects. The funder is in charge of the publication fees.

## Conflict of Interest

The authors declare that the research was conducted in the absence of any commercial or financial relationships that could be construed as a potential conflict of interest.

## Publisher’s Note

All claims expressed in this article are solely those of the authors and do not necessarily represent those of their affiliated organizations, or those of the publisher, the editors and the reviewers. Any product that may be evaluated in this article, or claim that may be made by its manufacturer, is not guaranteed or endorsed by the publisher.

## Abbreviations

GO, Gene Ontology; GSEA, gene set enrichment analysis; FDR, false discovery rate; AUC, area under the curve; C-index, concordance index; CI, confidence interval; ROC, receiver operating characteristic; ssGSEA, single-sample gene set enrichment analysis; TCGA, The Cancer Genome Atlas; HPA, human Protein Atlas; OS, overall survival; DSS, disease specific survival; Th1, type 1 T helper cells; Th2, type 2 T helper cells; Th17, type 1 T helper cells; Treg, regulatory T cell; iDCs, immature dendritic cells; NK cells, natural killer cells; Tem, T effector memory; Tcm, T central memory; Tfh, T follicular helper; Tgd, T gamma delta; aDC, activated DC; HNSCC, head and neck squamous cell carcinoma; OSCC, oral squamous cell carcinoma; PD-1, programmed death protein 1; PD-L1, programmed death protein ligand 1.
