# Corrigendum: DCBLD1 Overexpression is associated with a poor prognosis in head and neck squamous cell carcinoma

**DOI:** 10.3389/fimmu.2022.1099835

**Published:** 2022-11-25

**Authors:** Ling-ling Fu, Ming Yan, Min-Xian Ma, Yi Luo, Min Shao, Martin Gosau, Reinhard E. Friedrich, Tobias Vollkommer, Hong-chao Feng, Ralf Smeets

**Affiliations:** ^1^ Department of Oral and Maxillofacial Surgery, Guiyang Hospital of Stomatology, Guiyang, China; ^2^ Department of Oral and Maxillofacial Surgery, University Medical Center Hamburg-Eppendorf, Hamburg, Germany; ^3^ Department of Oral and Maxillofacial Surgery, Division of Regenerative Orofacial Medicine, University Medical Center Hamburg-Eppendorf, Hamburg, Germany

**Keywords:** DCBLD1, biomarker, immune infiltrates, prognosis, head and neck squamous cell carcinoma

In the published article, there was an error in affiliations 1 and 2. Instead of “^1^Department of Oral and Maxillofacial Surgery, University Medical Center Hamburg-Eppendorf, Hamburg, Germany, ^2^Department of Oral and Maxillofacial Surgery, Guiyang Hospital of Stomatology, Guiyang, China, “, it should be “^1^Department of Oral and Maxillofacial Surgery, Guiyang Hospital of Stomatology, Guiyang, China, ^2^Department of Oral and Maxillofacial Surgery, University Medical Center Hamburg-Eppendorf, Hamburg, Germany,”

The authors apologize for this error and state that this does not change the scientific conclusions of the article in any way. The original article has been updated.

## Publisher’s note

All claims expressed in this article are solely those of the authors and do not necessarily represent those of their affiliated organizations, or those of the publisher, the editors and the reviewers. Any product that may be evaluated in this article, or claim that may be made by its manufacturer, is notguaranteed or endorsed by the publisher.

