# Supplementary material for: DCBLD1 Overexpression Is Associated With a Poor Prognosis in Head and Neck Squamous Cell Carcinoma
Source: Front Immunol. 2022 Jul 1;13:939344. doi: 10.3389/fimmu.2022.939344 (PMC9283650; doi:10.3389/fimmu.2022.939344)
Supplement: Supplementary file 1 [file Table_1.docx]

| **Characteristics** | **Total(N)** | **Univariate analysis** | | **Multivariate analysis** | |
| --- | --- | --- | --- | --- | --- |
|  |  | **Hazard ratio (95% CI)** | ***P* value** | **Hazard ratio (95% CI)** | ***P* value** |
| Age(<=60 vs. >60) | 476 | 1.078 (0.763-1.524) | 0.670 |  |  |
| Gender(Female vs. Male) | 476 | 0.974 (0.656-1.447) | 0.897 |  |  |
| Clinical stage(Stage I&Stage II vs. Stage III&StageIV) | 462 | 1.151 (0.753-1.760) | 0.517 |  |  |
| T stage(T1&T2 vs. T3&T4) | 461 | 1.459 (0.988-2.153) | 0.057 | 2.289 (1.153-4.542) | **0.018** |
| N stage(N0 vs. N1&N2&N3) | 454 | 1.485 (1.044-2.112) | **0.028** | 1.046 (0.595-1.838) | 0.875 |
| M stage(M0 vs. M1) | 451 | 8.056 (2.527-25.680) | **<0.001** | 2.965 (0.602-14.600) | 0.182 |
| Primary therapy outcome(PD&SD&PR vs. CR) | 405 | 0.094 (0.061-0.146) | **<0.001** | 0.087 (0.050-0.151) | **<0.001** |
| Histologic grade(G1 vs. G2&G3) | 460 | 1.470 (0.828-2.610) | 0.189 |  |  |
| Lymphovascular invasion(No vs. Yes) | 326 | 1.658 (1.079-2.546) | **0.021** | 1.208 (0.711-2.053) | 0.484 |
| Lymphnode neck dissection(No vs. Yes) | 473 | 0.719 (0.465-1.114) | 0.140 |  |  |
| DCBLD1(High vs. Low) | 476 | 0.665 (0.468-0.944) | **0.023** | 0.502 (0.298-0.845) | **0.009** |
| Radiation therapy(No vs. Yes) | 424 | 0.740 (0.492-1.112) | 0.147 |  |  |
| Race(White vs. Black or African American&Asian) | 460 | 1.433 (0.847-2.425) | 0.180 |  |  |

Supplement table1. Association of clinicopathological characteristics with disease specific survival using univariate or multivariate Cox regression analysis
